# Robotic conservative treatment for prostatourethrorectal fistula: original technique step by step

**DOI:** 10.1590/S1677-5538.IBJU.2018.0584

**Published:** 2020-02-20

**Authors:** Michele Del Zingaro, Giovanni Cochetti, Gianluca Gaudio, Alberto Tiezzi, Alessio Paladini, Jacopo Adolfo Rossi de Vermandois, Ettore Mearini

**Affiliations:** 1 University of Perugia Urology Clinic Department of Surgical and Biomedical Sciences Perugia Italy Department of Surgical and Biomedical Sciences, Urology Clinic, University of Perugia, Santa Maria della Misericordia Hospital Piazzale Menghini, Perugia, Italy

## Abstract

**Purpose:**

Prostatourethrorectal fistula (PURF) is an uncommon complication resulting from surgery, radiation or trauma (1). The most common therapeutic management is transperineal surgery (1). Transabdominal approach is less used and limited to large fistulae needing cystectomy and rectal resection (1). The aim of this study was to show an original robotic technique of conservative treatment for PURF.

**Materials and Methods:**

A 75 years old man referred recurrent UTI, pneumaturia and urinary loss from rectum due to PURF arising after TURP performed after transvesical prostate adenomectomy. Cystogram, cystoscopy and MRI confirmed PURF. We used a robotic approach performing isolation, resection and suture of the fistulous tract on rectal and urethral side. Leak test was negative. We carried out an omental flap, positioned between rectum and prostatic urethra, and a temporary ileostomy without any bowel resection or urinary diversion.

**Results:**

Operative time was 210 minutes, estimated blood loss 50ml. Oral feeding was restored at 48 hours. Bladder catheter was removed on the 15th post-operative day. Post-operative cystogram was negative. Post-operative complications were ileus and urinary tract infection. Hospital stay was 10 days. At 6 months follow-up, before temporary ileostomy closure, cystoscopy showed a totally re-epithelised fovea, and cystogram and CT enterography were negative.

**Conclusions:**

Robotic conservative treatment of PURF seems to be safe and feasible (2, 3). Robotic approach allows accurate surgical dissection, through easier access to the rectal-prostatic plane, reducing the need for resection. To our knowledge, this is the first robotic conservative treatment for PURF reproducing the same steps of laparotomic approach with the advantages of minimally invasive technique (4).

## ABBREVIATIONS

PURF: prostatourethrorectal fistula
UTI: urinary tract infections
TURP: transurethral resection of the prostate
MRI: magnetic resonance imaging
